# Plasma Vitamin C, 25-Hydroxyvitamin D, and the Lactate-to-Albumin Ratio at PICU Admission in Survivors and Non-Survivors of Phoenix-Defined Pediatric Sepsis and Septic Shock: An Exploratory Prospective Cohort Study

**DOI:** 10.3390/pediatric18050132

**Published:** 2026-10-02

**Authors:** Jesús J. Martínez-García, Adrian Canizalez-Román, Ricardo Zatarain-Lopez, Marcel Antonio Cazarez-Aguilar, Martín Adrián Bolívar-Rodríguez, Luis Velazquez-Lopez, Carlos Alberto Osuna-Ramirez, Manuel A. Correa-Flores, Maria Fernanda Garcia-de Leon, Erika Acosta-Smith, Jorge Velazquez-Roman, Hector Flores-Villaseñor, Uriel A. Angulo-Zamudio, Nidia León-Sicairos

**Affiliations:** 1 Pediatric Hospital of Sinaloa, Culiacán 80200, Sinaloa, Mexico; jjmtz64@hotmail.com (J.J.M.-G.); luis.velazquez.lopez@hotmail.com (L.V.-L.); carlos.osunar@gmail.com (C.A.O.-R.); fernanda.gardeleon@gmail.com (M.F.G.-d.L.); 2 School of Medicine, Autonomous University of Sinaloa, Culiacán 80019, Sinaloa, Mexico; erika.acosta@uas.edu.mx (E.A.-S.); jorgevelazquezroman@uas.edu.mx (J.V.-R.); floreshector@uas.edu.mx (H.F.-V.); uriel.zamudio@uas.edu.mx (U.A.A.-Z.); 3 The Women’s Hospital, Secretariat of Health, Culiacán 80020, Sinaloa, Mexico; 4 Centro de Investigación y Docencia en Ciencias de la Salud (CIDOCS), Autonomous University of Sinaloa, Culiacán 80030, Sinaloa, Mexico; ricardo.zatarainn@gmail.com (R.Z.-L.); marcelcazarez@hotmail.com (M.A.C.-A.); bolivarmartin64@hotmail.com (M.A.B.-R.); 5 IMSS Bienestar Legaria Pediatric Hospital, Mexico City 11260, Mexico; correautip@yahoo.com.mx

**Keywords:** pediatric sepsis, Phoenix criteria, vitamin C, ascorbic acid, 25-hydroxyvitamin D, lactate-to-albumin ratio, pediatric intensive care unit, mortality

## Abstract

Background: Vitamin C, 25-hydroxyvitamin D (25[OH]D), and the lactate-to-albumin ratio (LAR) have been proposed as prognostic markers in sepsis. However, pediatric data are limited, and these markers have not been evaluated together in a cohort classified according to the Phoenix criteria. We quantified these markers at pediatric intensive care unit (PICU) admission and assessed their unadjusted association with mortality. Methods: This single-center prospective cohort study followed the STROBE statement. We enrolled consecutive children aged 1 month to 17 years with suspected infection and a Phoenix Sepsis Score of at least 2; septic shock required at least one cardiovascular point. Blood was sampled within 1 h of admission. We analyzed ascorbate by ultra-high-performance liquid chromatography coupled with mass spectrometry in light-protected heparin plasma. The endpoint was pre-discharge PICU mortality. We expressed differences as Hodges–Lehmann estimates with exact 95% confidence intervals (CIs), and discrimination as the area under the receiver operating characteristic curve (AUC). The five principal biomarker comparisons were subjected to Benjamini–Hochberg false discovery rate (FDR) control. We fitted no multivariable model (11 events). Results: Of the 37 children enrolled, 33 (89.2%) had septic shock, and the mortality rate was 29.7%. Non-survivors had lower plasma vitamin C concentrations (51.1 vs. 73.8 umol/L; difference −17.0, 95% CI −28.4 to −5.7, *p* = 0.028, q = 0.141, AUC 0.73, 95% CI 0.55–0.90), and mortality declined across ascending vitamin C tertiles, although the gradient was not statistically significant (*p* = 0.058). No patient had hypovitaminosis C (<23 umol/L). We did not detect an association between 25(OH)D, lactate, albumin, or the LAR and mortality (all *p* ≥ 0.18; AUC 0.50–0.64), and the LAR was collinear with lactate (rho = 0.95). The weight-for-age z-score correlated with vitamin C (rho = 0.39, *p* = 0.033). Conclusions: In this small cohort, non-survivors had lower plasma vitamin C concentrations within the replete range in unadjusted analysis; this difference did not persist after FDR control or in the subgroup with evaluable nutritional status, and nutritional confounding cannot be excluded. No association with mortality was detected for 25(OH)D, lactate, albumin, or the LAR. These findings are hypothesis-generating, do not establish prognostic value, and require multicenter confirmation.

## 1. Introduction

Ascorbic acid, also known as vitamin C, is a water-soluble antioxidant that acts as a cofactor for enzymes involved in protein and hormone synthesis, energy metabolism, and gene transcription regulation [1]. Humans cannot synthesize ascorbic acid endogenously and must obtain it through diet [1,2]. Plasma ascorbic acid concentrations below 23 μmol/L are considered hypovitaminosis C, while concentrations below 11 μmol/L are considered vitamin C deficiency [3,4]. Low concentrations have been reported in conditions characterized by increased oxidative stress, including sepsis and acute respiratory distress syndrome [5,6].

Vitamin D contributes to calcium homeostasis, bone maturation, cardiovascular function, and innate and adaptive immunity [7,8]. Pediatric critical care studies have commonly classified serum 25-hydroxyvitamin D (25[OH]D) concentrations as sufficient (>30 ng/mL), insufficient (20–30 ng/mL), or deficient (<20 ng/mL) [9,10]. These cut-offs derive from the 2011 Endocrine Society guideline [11]; the 2024 Endocrine Society guideline no longer endorses specific 25(OH)D concentrations to define sufficiency, insufficiency, or deficiency [12]. These categories are used here only as historical cut-offs to allow comparison with previous pediatric studies. Vitamin D deficiency has been associated with higher infection rates, greater disease severity, longer PICU length of stay, and higher mortality in some PICU cohorts, although the evidence is heterogeneous [13,14,15,16,17].

In addition to micronutrients, serum lactate and albumin are well-established markers of tissue hypoperfusion and inflammatory–nutritional status, respectively. Elevated lactate levels at admission and hypoalbuminemia have both been associated with mortality in critically ill children [18,19,20,21]. The lactate-to-albumin ratio (LAR), which combines these two parameters, has been proposed as an early indicator of severity in the PICU, with a frequently cited threshold above 0.5 derived from a large multicenter PICU cohort [22,23]; higher optimal cut-offs (approximately 1.1–1.2) have been reported in a prospective pediatric cohort of severe sepsis [24].

This study addresses three gaps in the current evidence. First, the literature on vitamin C in pediatrics is limited to small cohorts from Switzerland, the United Kingdom, Australia, the United States, and China, in which 10–47% of children had hypovitaminosis C [2,25,26,27]. The prognostic relevance of ascorbate concentrations within the adequate range has not been examined, and no Latin American cohort of children with sepsis has been reported. Second, researchers have primarily evaluated the LAR in retrospective cohorts and in populations with hyperlactatemia and high mortality. For instance, a prospective Vietnamese cohort had a 63.4% mortality rate, and the optimal LAR cutoff value was 1.84 [28]. Therefore, it remains unclear whether the frequently cited threshold of 0.5 retains any discriminatory value when admission lactate levels are normal. This threshold has never been externally validated in prospectively enrolled children classified according to the 2024 Phoenix criteria. Third, vitamin C, 25(OH)D, lactate, albumin, and the LAR have not been analyzed together in a prospectively enrolled pediatric cohort, so their relative performance has only been inferred across studies that differ in population, timing, and assay methods.

This exploratory, hypothesis-generating study had a twofold objective: first, to describe plasma concentrations of vitamin C, 25(OH)D, lactate, albumin, and the LAR at admission to the pediatric intensive care unit (PICU), and second, to examine their association with all-cause mortality among children with sepsis and septic shock, as defined by the Phoenix criteria [29,30]. Given the planned sample size, the study was not designed to estimate independent prognostic value, to compare the discriminatory power of competing biomarkers, or to develop or validate predictive models.

## 2. Materials and Methods

### 2.1. Study Design and Setting

This single-center prospective observational cohort study measured biomarkers at admission (exposure) and ascertained survival status at discharge from the pediatric intensive care unit (PICU; outcome). The study was conducted in the PICU of the Pediatric Hospital of Sinaloa in Culiacan, Sinaloa, Mexico, from 1 January to 31 December 2024. Reporting followed the Strengthening the Reporting of Observational Studies in Epidemiology (STROBE) statement for cohort studies [31,32]. A completed STROBE checklist for cohort studies is provided as a Supplementary Table S1.

### 2.2. Participants and Eligibility Criteria

We enrolled consecutive patients aged 1 month to 17 years who were admitted to the PICU with suspected or confirmed infection. Inclusion criteria required a Phoenix Sepsis Score of at least 2, which defines sepsis in children with suspected infection. Septic shock was defined as sepsis with at least one cardiovascular point, as specified in the Phoenix criteria [29,30]. We excluded patients receiving ascorbic acid or vitamin D supplements and immunocompromised patients.

We obtained written informed consent from the parents or legal guardians of all participants before enrollment. In addition, we obtained assent from children aged 7 years or older whenever their clinical condition allowed. Blood for the study biomarkers was drawn during the same venipuncture as the routine admission laboratory panel, so no additional venipuncture was performed for research purposes. We coded and de-identified all data before analysis.

#### Cardiovascular Component

Cardiovascular component: Because the distinction between sepsis and septic shock rests exclusively on the cardiovascular subscore, this component is reported explicitly. Phoenix cardiovascular points were assigned as follows: one point for a single infusion of vasoactive agents, and two points for two or more infusions; one point for a lactate level between 5.0 and 10.9 mmol/L, and two points for a lactate level of at least 11 mmol/L. Age-adjusted mean arterial pressure was not prospectively recorded and was therefore not included in the cardiovascular subscore. Because vasoactive support and lactate levels already conferred at least one cardiovascular point in all patients classified this way, omitting mean arterial pressure could only underclassify, not overclassify, septic shock. This omission is addressed in the Section 4.1.

One patient had a recorded total Phoenix Sepsis Score of 0 even though two vasoactive infusions, invasive mechanical ventilation, and renal replacement therapy were administered simultaneously. According to the Phoenix scale, two vasoactive infusions alone yield two cardiovascular points, so the patient could not have a total score of zero. The pSOFA score of the same patient was also recorded as 0, which is equally incompatible with the documented organ support; the most likely explanation is that the severity scores were not entered for this patient and a default value of zero was recorded. We therefore treated the recorded value as a data-entry error rather than as evidence that the patient did not have sepsis. We retained the patient and classified the episode as septic shock. A sensitivity analysis that excludes this patient is presented in Section 3.7. The total Phoenix score recorded on the case report form was not recalculated from its four component subscores because the respiratory, coagulation, and neurological components were not recorded separately. This constraint is acknowledged in the Section 4.1. The cohort therefore comprises both sepsis and septic shock, and this distinction is retained throughout.

### 2.3. Outcome

The outcome was all-cause mortality occurring before discharge from the PICU (PICU mortality). Because no time-to-event variables were recorded, neither 28-day nor in-hospital mortality could be reported, and no survival analysis was performed. Survival status was ascertained for every enrolled patient, and follow-up ended at PICU discharge.

### 2.4. Clinical Variables

Demographic, clinical, and laboratory variables were prospectively recorded. Severity was quantified using the Phoenix Sepsis Score, the Pediatric Risk of Mortality (PRISM III) Score, and the Pediatric Sequential Organ Failure Assessment (pSOFA) Score. These scores were obtained within the first 12 h after admission. The number of vasoactive agents, the vasoactive–inotropic score, the need for and duration of invasive mechanical ventilation, the need for renal replacement therapy, and the length of stay in the PICU were also recorded. Nutritional status was assessed using weight-for-age z-scores. According to the WHO Child Growth Standards (2006) [33], malnutrition was defined as a weight-for-age z-score below −2 and severe malnutrition as a z-score below −3. Because the WHO Child Growth Standards cover children aged 0 to 60 months, the weight-for-age analysis was intentionally restricted to children within this age range (Section 3.5).

#### 2.4.1. Timing of Sample Collection in Relation to Treatment

We collected blood for all biomarkers within the first hour after admission to the PICU, using the same venipuncture as the routine admission laboratory panel. We sampled before administering exogenous albumin or parenteral nutrition. However, patients had already received initial fluid resuscitation and, in most cases, vasoactive support in the emergency department or referring unit before admission to the PICU. We did not record the volume of fluid administered before sampling and could not adjust for it. Because hemodilution reduces albumin and micronutrient concentrations, unmeasured differences in pre-admission fluid load could lower vitamin C, 25(OH)D, and albumin concentrations simultaneously, thereby biasing their associations with mortality in the same direction.

#### 2.4.2. Unavailable Variables

Pre-existing comorbidities, the anatomical origin of the infection, microbiological culture results, the volume and type of resuscitation fluids, exogenous albumin administration, and the type and timing of nutritional support were not recorded and could not be analyzed. Their absence is acknowledged in the Section 4.1. Table 1 summarizes available variables related to organ support and treatment (invasive mechanical ventilation, number of vasoactive agents, vasoactive–inotropic score, renal replacement therapy, and corticosteroid administration).

### 2.5. Biomarker Measurement and Preanalytical Handling

#### 2.5.1. Biological Matrices

Ascorbate was measured in lithium heparin plasma; 25(OH)D, albumin, and procalcitonin were measured in serum; and lactate was measured in whole arterial blood using a blood gas analyzer. Because heparinized plasma yields higher ascorbate concentrations than serum, we specify the matrix when reporting ascorbate results.

Blood for the analysis of vitamin C and 25(OH)D was drawn within the first hour after admission to the PICU into separate, light-protected (amber) tubes. The handling of the vitamin C sample is described in detail below. Vitamin C was quantified using ultra-high-performance liquid chromatography coupled with mass spectrometry (UPLC-MS). Serum 25(OH)D was quantified using a fluorescence immunoassay (Wondfo Finecare FIA Meter III Plus). Procalcitonin was measured by enzyme-linked immunosorbent assay. Arterial blood lactate was determined using an enzymatic lactate oxidase method on a blood gas analyzer (ABL90 FLEX, Radiometer, Brønshøj, Denmark; range: 0.1–30.0 mmol/L, coefficient of variation < 3%). Serum albumin was measured using the bromocresol green colorimetric method on a Cobas c311 analyzer (Roche Diagnostics, Rotkreuz, Switzerland; range: 1.0–6.0 g/dL; analytical imprecision < 2.5%). The LAR was calculated by dividing arterial lactate (mmol/L) by albumin (g/dL).

#### 2.5.2. Preanalytical Handling of the Vitamin C Sample

Ascorbate is unstable ex vivo: it oxidizes rapidly at room temperature, at alkaline pH, and on exposure to light. Valid measurement therefore requires protection from light at all times, cold handling from the moment of collection, rapid centrifugation while the sample is cold, and analysis within the interval during which ascorbate remains stable under refrigeration [34,35,36,37]. The protocol used in this study met these requirements and is described in full below.

#### 2.5.3. Collection, Transport, and Processing

Blood for vitamin C analysis was collected within the first hour after admission to the PICU, from the same venipuncture used for the routine admission laboratory panel, into a light-protected lithium heparin tube. The tube was immediately placed on ice at 0–4 °C and transported without delay to the hospital’s clinical laboratory. The unit retained no samples. On arrival, the samples were centrifuged in a 4 °C-cooled centrifuge, and the separated plasma was stored at 2–8 °C, continuously protected from light, until analysis. All samples were analyzed within 48 h of collection. No aliquots were frozen or subjected to a freeze–thaw cycle. Laboratory staff performed the analyses without knowledge of the patient’s clinical course or outcome.

All conditions identified in the literature as essential for ascorbate determination were met. Samples were protected from light throughout the process, from collection to analysis. They were kept refrigerated at all times, centrifuged immediately while cold, and analyzed within the interval during which ascorbate is preserved in heparinized, refrigerated, and light-protected samples. Lithium heparin was used because ascorbate is stable in cold-stored, heparinized blood, whereas it degrades rapidly in EDTA under the same conditions [38]. In addition, heparinized plasma provides higher and more reproducible ascorbate values than serum [35]. Because all samples were analyzed within 48 h and none were archived, no sample underwent prolonged storage or freeze–thaw cycles, which require acid stabilization and ultra-low-temperature storage. All samples were collected, transported, and processed using identical procedures, irrespective of the patient’s clinical outcome. Outcomes were unknown at the time of analysis; handling conditions were therefore uniform for survivors and non-survivors.

#### 2.5.4. Analytical Method

As part of its routine diagnostic service, the hospital’s central clinical laboratory quantified vitamin C using UPLC-MS in accordance with the laboratory’s established procedure. Results were expressed to one decimal place (mg/dL). Data on internal standard values, calibration range, lower limit of quantification, and intra- and inter-assay coefficients of variation were unavailable. These parameters are in the laboratory’s internal method validation records, which we did not have access to.

#### 2.5.5. Unit Conversion

Vitamin C was measured in milligrams per deciliter (mg/dL) and converted to micromoles per liter (µmol/L) using the molar mass of ascorbic acid (176.12 g/mol), such that 1 mg/dL = 56.78 µmol/L.

#### 2.5.6. Reference Thresholds

Vitamin C: Hypovitaminosis < 23 umol/L, deficiency < 11 umol/L [3,4]. 25(OH)D: deficiency < 20 ng/mL, insufficiency 20–30 ng/mL, and sufficiency > 30 ng/mL. These categories are commonly used in previous pediatric studies [9,10] and correspond to the historical 2011 Endocrine Society cut-offs [11]; they are not current Endocrine Society criteria, because the 2024 guideline no longer endorses specific 25(OH)D thresholds [12]. They are used for descriptive comparison only. LAR: A threshold of 0.5 was applied for descriptive purposes only, as it was neither derived nor validated in this cohort [22,23].

### 2.6. Statistical Analysis

The analysis was descriptive and hypothesis-generating. Because the study included all eligible patients admitted during a fixed 12-month period, we did not perform a priori sample size calculation. A post hoc calculation indicated that, with 26 survivors and 11 non-survivors, a two-sided significance level (a) of 0.05, and 80% power, the minimum detectable standardized effect size was Cohen’s d = 1.04. The study could therefore detect only very large effects. This calculation is reported as a limitation, not as a post hoc justification. No primary hypothesis and no statistical analysis plan were specified in advance. The five principal biomarker comparisons (vitamin C, 25(OH)D, lactate, albumin, and the LAR), which correspond to the study objectives, were defined as the family of comparisons for multiplicity control; all other comparisons were exploratory.

Distribution assumptions were assessed using the Shapiro–Wilk test. Continuous variables were summarized as medians with interquartile ranges (IQR), or as means with standard deviations (SD) when normality was confirmed. Between-group comparisons used the Mann–Whitney U test for non-normally distributed variables and Welch’s *t*-test for normally distributed variables. Fisher’s exact test (2 × 2) or a Monte Carlo exact test with 100,000 replicates (rxc tables) was used to compare categorical variables, because the expected counts per cell were fewer than 5.

Effect sizes were reported with 95% confidence intervals (CIs): the Hodges–Lehmann median difference with distribution-free exact confidence limits derived from the Mann–Whitney statistic, Cliff’s delta for rank-based comparisons, the mean difference with Welch’s CI (for normally distributed variables), and Wilson’s CI (for proportions). Discrimination for mortality was quantified as the area under the receiver operating characteristic curve (AUC) with a 95% CI obtained by bootstrapping with 4000 resamples. Because of the small number of deaths (approximately one per candidate predictor), a conventional multivariable logistic model could not be fitted. Instead, an unadjusted Firth-penalized logistic regression was used to obtain odds ratios with 95% CIs; this method is less biased with sparse data. Firth-penalized odds ratios were reported for all five principal biomarkers, not only for those with nominally statistically significant unadjusted comparisons, so that the presentation is independent of the outcome. The confidence limits were profile-penalized likelihood limits. These estimates are presented for exploratory purposes only and are not described as independent or adjusted associations.

The Benjamini–Hochberg false discovery rate [39] was used to control multiplicity in the five principal biomarker comparisons. Both unadjusted *p*-values and q-values are presented. AUC values were not formally compared, and the incremental value relative to the Phoenix or PRISM III scores was not evaluated. Calibration was not assessed, and no internal or external validation was performed, because the small sample size would render such analyses uninterpretable. Missing data were minimal (serum calcium and phosphorus were missing for one patient each, representing 2.7%). No imputation was performed, and analyses were based on complete cases. The denominators for each comparison are reported. Two sensitivity analyses were performed in the revised analysis, in response to issues identified during peer review: one that excluded the patient with an internally inconsistent Phoenix record, and one that restricted the analysis to patients who met the criteria for septic shock. Both used the same tests as the main analysis. The cross-classification of vitamin C and 25(OH)D status was a post hoc exploratory analysis and is reported only in the Supplementary Material (Supplementary Table S2 and Supplementary Figure S3). The data were analyzed using IBM SPSS Statistics for Windows, version 27 (IBM Corp., Armonk, NY, USA), and SigmaPlot (v. 15, Systat Software, San Jose, CA, USA). Before the revised analysis, all variables reported in Table 1 and Table 2 were rechecked against the case report forms and laboratory reports, derived variables (unit conversions, 25(OH)D categories, the LAR, and weight-for-age z-scores) were recalculated from the verified primary variables, and all analyses were re-run on the verified database.

## 3. Results

### 3.1. Cohort Characteristics and Application of the Phoenix Criteria

Participant flow: During the 12-month enrollment period, 229 children were admitted to the PICU. Of these, 40 were screened for suspected or confirmed infection and evaluated against the Phoenix-based eligibility criteria. Three were excluded because they were immunocompromised, and 37 were included in the analysis. Survival status at PICU discharge was available for all 37 patients (Supplementary Figure S1).

The median age was 10 months (interquartile range [IQR] 3–24; range 1–204 months), and 23 patients (62.2%) were male. Under the Phoenix criteria, 33 patients (89.2%; 95% CI 75.3–95.7) met the definition of septic shock, and four (10.8%) met the criteria for sepsis without shock. Eleven patients died before PICU discharge, giving an all-cause PICU mortality of 29.7% (95% CI 17.5–45.8). Cardiovascular points were assigned to the 33 patients who received vasoactive support (one agent: *n* = 12, one point; two or more agents: *n* = 21, two points) and to five patients with a lactate level > 5 mmol/L, all of whom were already receiving vasoactive support. The median cardiovascular subscore was 2 (IQR 1–2); we found no difference between survivors and non-survivors (*p* = 0.554). The four children without shock had normal lactate levels (0.6–2.1 mmol/L); three survived, and one died. The median follow-up time, defined as the interval from PICU admission to death or discharge, was 22 days (IQR 11–52 days). Table 1 shows the baseline characteristics of survivors and non-survivors.

### 3.2. Plasma Vitamin C Levels

Plasma vitamin C concentrations ranged from 0.8 to 2.0 mg/dL (45.4 to 113.6 µmol/L). No patient had a concentration at or below the hypovitaminosis threshold of 23 µmol/L (0/37; 95% CI 0–9.4%), and the lowest observed value (45.4 µmol/L) was approximately twice that threshold. This analysis therefore evaluates differences in vitamin C concentration within the adequate range, above the hypovitaminosis threshold, rather than the relationship between vitamin C deficiency and mortality.

**Figure 1:**
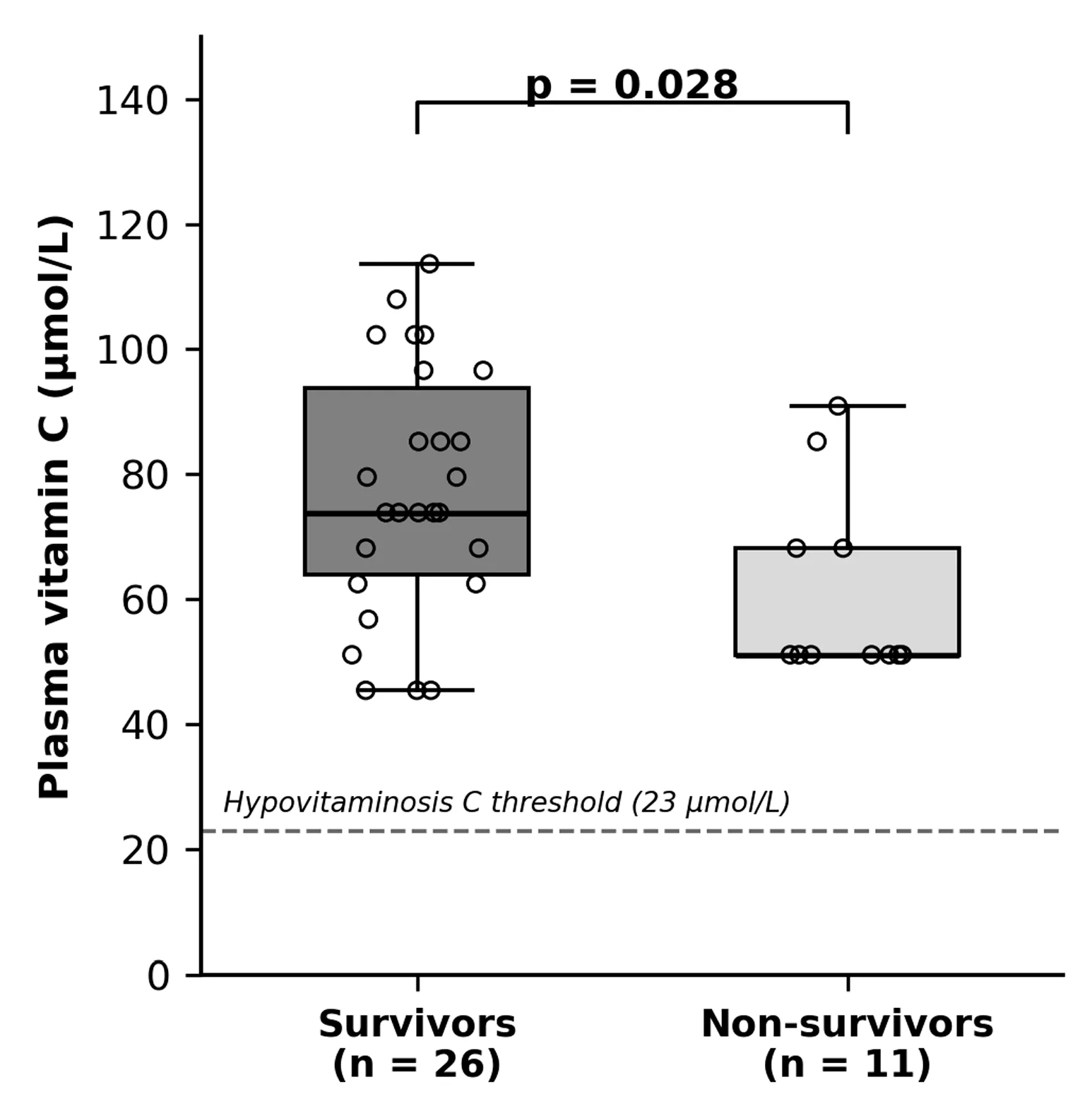
Plasma vitamin C concentrations at PICU admission in survivors and non-survivors. The box plots show medians and interquartile ranges with overlaid individual data points. The comparison used the Mann–Whitney U test (*p* = 0.028; q = 0.141 after Benjamini–Hochberg correction). The dashed line marks the hypovitaminosis threshold of 23 umol/L, which lies below the entire observed range (lowest observed value, 45.4 umol/L). Concentrations were converted from mg/dL using the molar mass of ascorbic acid (1 mg/dL = 56.78 umol/L).

Non-survivors had lower vitamin C concentrations than survivors, with a median of 51.1 µmol/L (IQR 51.1–68.1; 0.90 mg/dL, IQR 0.90–1.20) compared with 73.8 µmol/L (IQR 63.9–93.7; 1.30 mg/dL, IQR 1.12–1.65). The Hodges–Lehmann median difference was −17.0 µmol/L (95% CI −28.4 to −5.7), Cliff’s delta was −0.46, and the Mann–Whitney U was 209.0 (*p* = 0.028). Discrimination for mortality was AUC 0.73 (95% CI 0.55–0.90). The unadjusted Firth-penalized odds ratio was 0.63 per 10 µmol/L increase (95% CI 0.37–0.94; *p* = 0.020). After Benjamini–Hochberg correction across the five principal biomarker comparisons, the association was no longer statistically significant (q = 0.141). Because vitamin C was recorded to one decimal place in mg/dL, corresponding to a measurement granularity of 5.7 µmol/L, numerous ties occurred (eight of the 37 patients shared the value 0.9 mg/dL, seven of whom were among the 11 non-survivors), which limits the resolution of rank-based comparisons (Figure 1). Table 2 presents the effect estimates, discrimination, and FDR-corrected q-values for the five principal biomarker comparisons.

**Figure 2:**
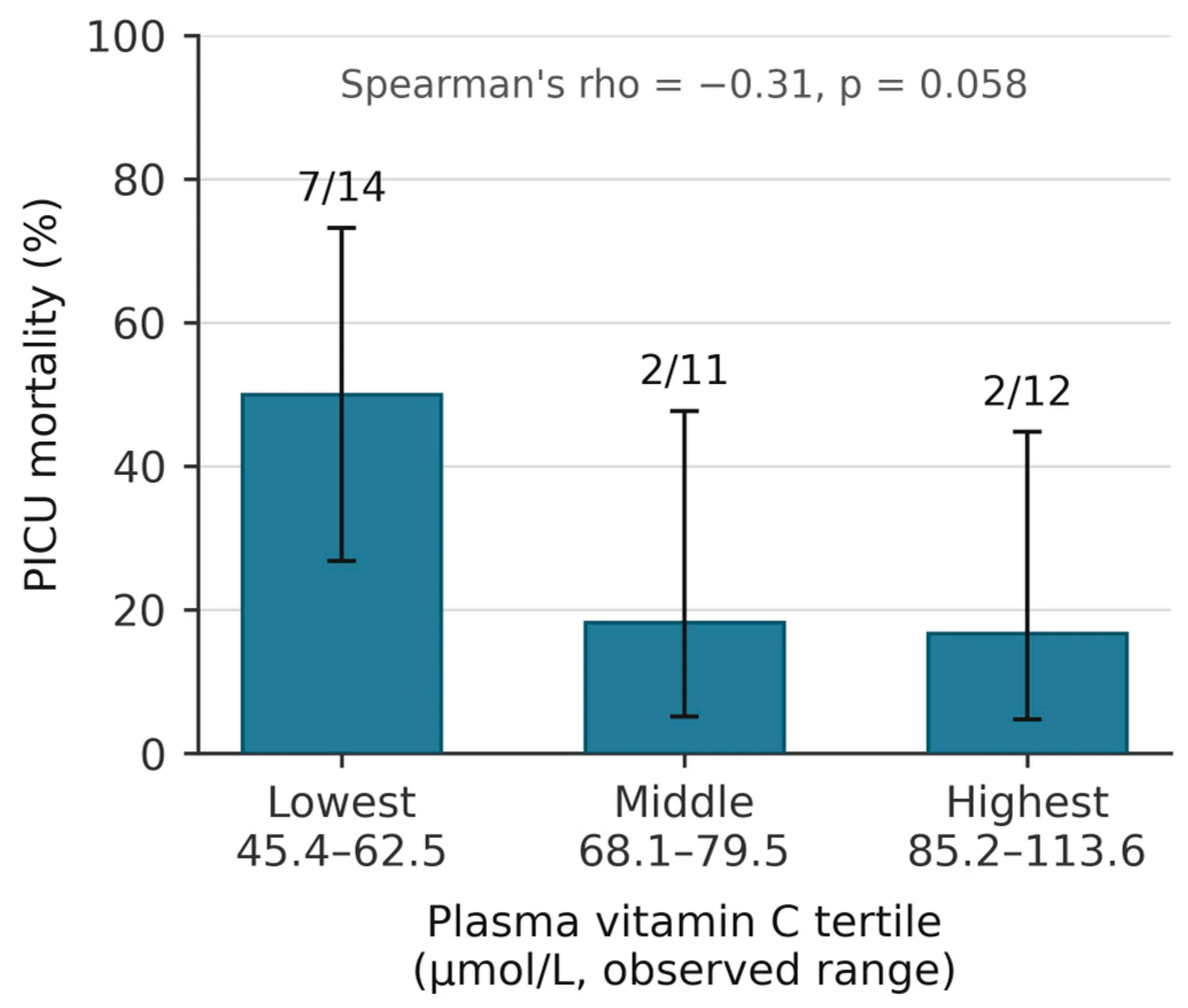
PICU mortality by plasma vitamin C tertile. The entire range lies above the hypovitaminosis threshold of 23 umol/L. The numbers above the bars indicate deaths relative to the total. The gradient across tertiles was not statistically significant (Spearman’s rho = −0.31, *p* = 0.058; vital status coded as 1 = death).

Two exploratory observations characterized the distribution of vitamin C in relation to mortality. First, mortality decreased across ascending vitamin C tertiles: seven of 14 (50.0%) in the lowest tertile (observed range 45.4–62.5 µmol/L), two of 11 (18.2%) in the middle tertile (68.1–79.5 µmol/L), and two of 12 (16.7%) in the highest tertile (85.2–113.6 µmol/L; Figure 2). The gradient across tertiles was not statistically significant (Spearman’s rho = −0.31, *p* = 0.058). Because the measurement granularity creates gaps between tertiles, the boundaries are given as observed ranges rather than cutoff values. For completeness, the rank correlation between untransformed vitamin C concentration and vital status was rho = −0.37 (*p* = 0.025); this unadjusted exploratory statistic does not test for trend. Second, vitamin C correlated inversely with the pSOFA score (Spearman’s rho = −0.39, *p* = 0.017); no correlation was detected with PRISM III (rho = −0.01, *p* = 0.946), the vasoactive–inotropic score (rho = −0.06, *p* = 0.742), procalcitonin (rho = −0.18, *p* = 0.277), albumin (rho = −0.07, *p* = 0.696), or lactate (rho = −0.16, *p* = 0.350). These observations were exploratory and were not corrected for multiplicity.

### 3.3. 25-Hydroxyvitamin D

**Figure 3:**
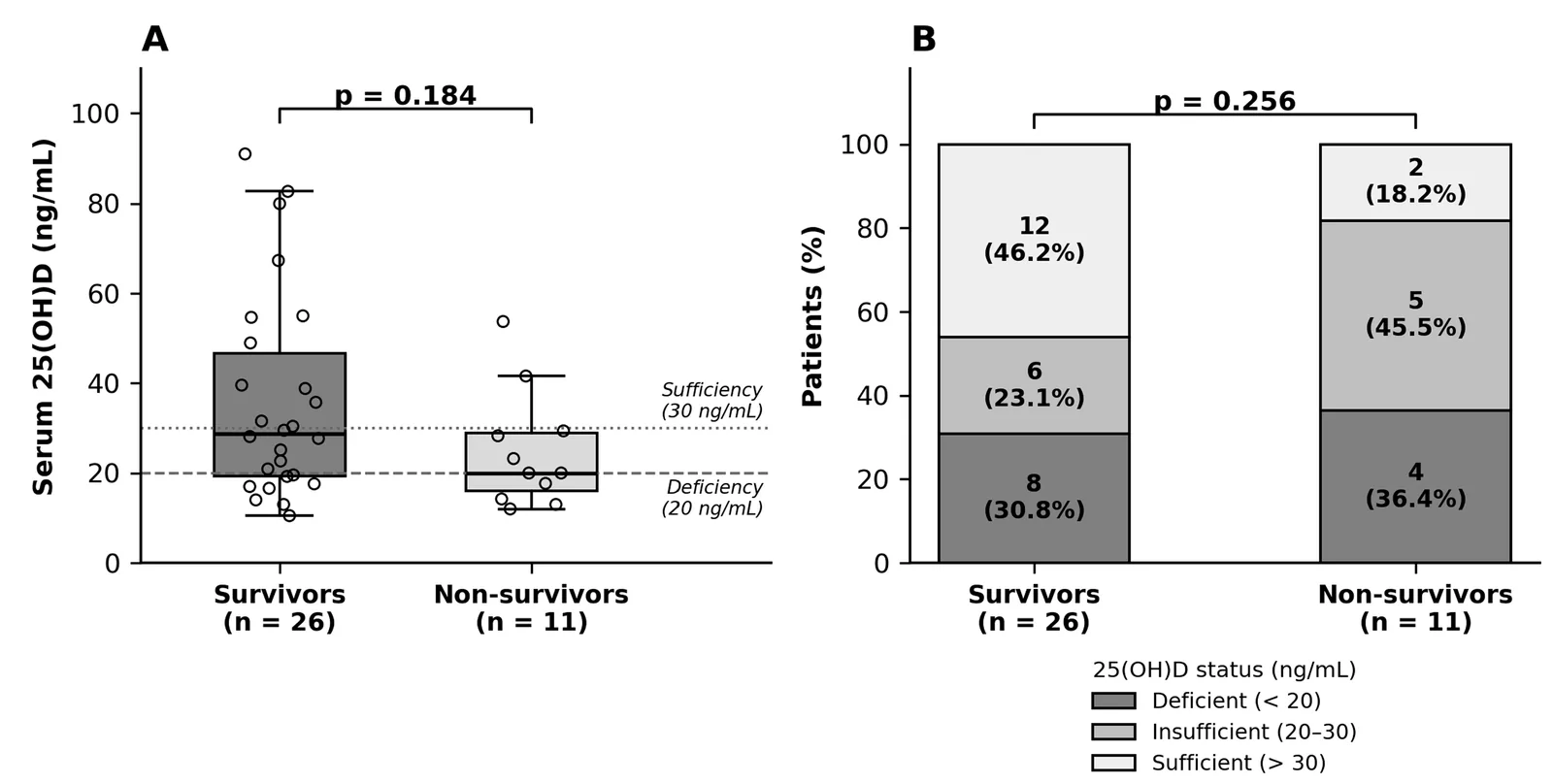
Serum 25-hydroxyvitamin D concentrations at PICU admission in survivors and non-survivors. (**A**) 25(OH)D concentrations. Box plots show medians and interquartile ranges, with individual data points overlaid. The Mann–Whitney U test yielded *p* = 0.184. The dashed and dotted horizontal lines indicate the historical cut-offs of 20 ng/mL and 30 ng/mL commonly used in previous pediatric studies (not current Endocrine Society criteria). (**B**) Distribution of 25(OH)D categories by vital status. The bars show the percentage of each group, with absolute numbers and percentages within each segment (Monte Carlo exact test, 100,000 replicates, *p* = 0.256).

The median 25(OH)D concentration was 28.8 ng/mL (IQR 19.3–46.6) in survivors and 20.0 ng/mL (IQR 15.9–28.8) in non-survivors. The Hodges–Lehmann difference was −6.9 ng/mL (95% CI −18.8 to 2.4; *p* = 0.184; AUC 0.64, 95% CI 0.45–0.82; Figure 3A). A 25(OH)D concentration < 20 ng/mL) was present in 12 patients (32.4%; 95% CI 19.6–48.5): eight of 26 survivors (30.8%) and four of 11 non-survivors (36.4%). We did not detect a difference between groups in the distribution across the three historical 25(OH)D categories (>30 ng/mL, 12 vs. 2; 20–30 ng/mL, 6 vs. 5; <20 ng/mL, 8 vs. 4; Monte Carlo exact test, *p* = 0.256; Figure 3B).

### 3.4. Lactate, Albumin, and Lactate-to-Albumin Ratio

**Figure 4:**
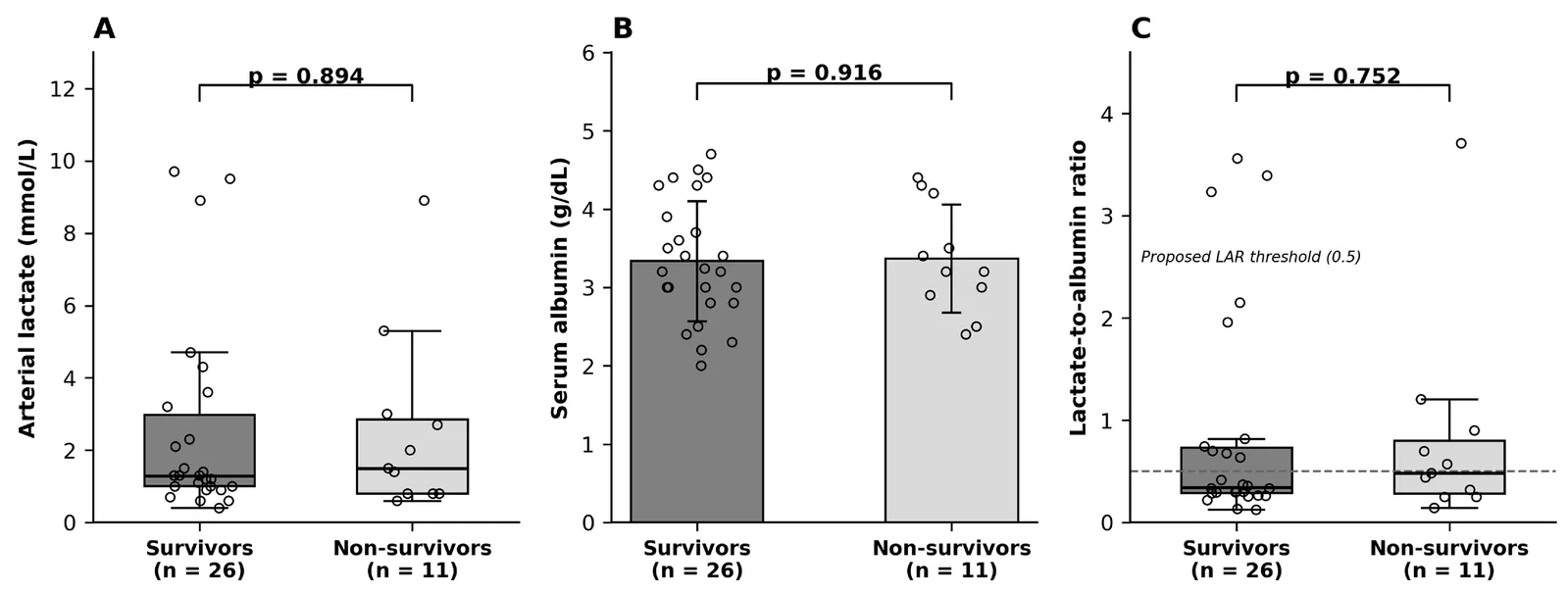
(**A**) arterial lactate, (**B**) serum albumin, and (**C**) the lactate-to-albumin ratio in survivors and non-survivors. Panels (**A**,**C**) are box plots showing medians and interquartile ranges with individual data points overlaid (Mann–Whitney U test), and panel (**B**) is a bar graph showing means and standard deviations with individual data points overlaid (Welch’s *t*-test). The dashed line in panel (**C**) marks the proposed threshold for the lactate-to-albumin ratio (LAR) of 0.5. This threshold was applied solely for descriptive purposes and was neither derived nor validated in this cohort.

**Figure 5:**
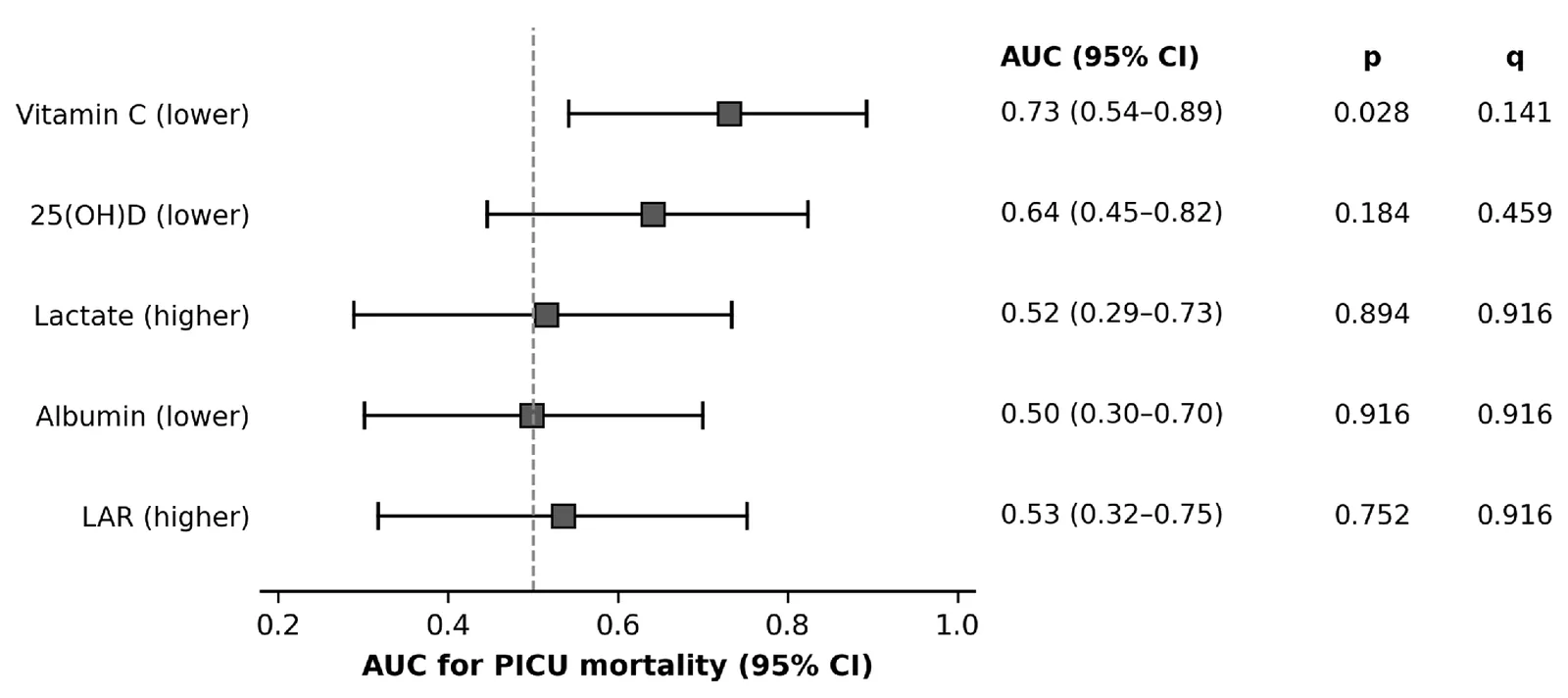
Discrimination of the five principal biomarkers for PICU mortality, expressed as the area under the receiver operating characteristic curve (AUC) with 95% confidence intervals obtained by bootstrapping (4000 resamples, fixed seed). Grey squares indicate the point estimate of the AUC for each biomarker, and horizontal lines with end caps indicate the 95% confidence interval. The vertical dashed line marks an AUC of 0.50, which corresponds to no discrimination (chance level). The direction of each comparison is indicated in parentheses and denotes the hypothesized adverse direction, not the observed one. No between-group difference was detected for albumin or lactate. Unadjusted *p*-values and Benjamini–Hochberg q-values are shown on the right. Only vitamin C has a confidence interval that excludes 0.50; however, its association is not statistically significant after multiple-comparison correction.

The median arterial lactate was 1.30 mmol/L (IQR 1.00–2.98) in survivors and 1.50 mmol/L (IQR 0.80–2.85) in non-survivors (*p* = 0.894; AUC 0.52, 95% CI 0.30–0.73). The mean serum albumin was 3.34 g/dL (SD 0.77) in survivors and 3.36 g/dL (SD 0.69) in non-survivors; the mean difference (non-survivors minus survivors) was +0.03 g/dL (95% CI −0.51 to 0.56; Welch’s *t*-test, *p* = 0.916; AUC 0.50, 95% CI 0.31–0.69). The median LAR was 0.35 (IQR 0.29–0.73) in survivors and 0.48 (IQR 0.29–0.80) in non-survivors (*p* = 0.752; AUC 0.53, 95% CI 0.32–0.74). Fifteen patients (40.5%) had an LAR above the previously proposed threshold of 0.5. Mortality was 33.3% (5/15) among those above the threshold and 27.3% (6/22) among those at or below it (Fisher’s exact test, *p* = 0.728; Figure 4). Table 2 summarizes the five principal comparisons, and Figure 5 illustrates them.

Two children were recorded as having scores of 0 (*p* < 0.001), but with albumin at only rho = −0.34 (*p* = 0.038). Second, no discrimination for mortality was detected when the analysis was restricted to the 13 children whose admission lactate exceeded 2 mmol/L (four deaths; AUC 0.50, *p* = 1.000); this restricted analysis addressed the possibility that the inclusion of normolactatemic patients diluted the association. With only four events, the subgroup analysis is inconclusive; it is reported to document that no signal was detected, not to establish the absence of an effect. Most of the cohort had normal admission lactate (median 1.30 mmol/L; 11 of 37 patients had values above 2.5 mmol/L), and albumin concentrations were virtually identical between groups; neither component of the ratio distinguished survivors from non-survivors in this cohort.

### 3.5. Nutritional Status

Weight-for-age z-scores were calculated using the WHO Child Growth Standards (2006) from age, sex, and weight [33]. Five of the 37 children were older than 60 months, the upper age limit of these standards (72, 156, 168, 192, and 204 months); four of them were older than 120 months, beyond which no WHO weight-for-age reference is available [40]. The 72-month-old child could have been assessed with the WHO 2007 Growth Reference [40], but the analysis was intentionally restricted to the WHO 2006 standards to use a single reference; this child was therefore also excluded. Of the remaining 32 records, two contained anthropometric values that were biologically implausible for the recorded age (a 2-month-old infant recorded at 13 kg and 110 cm, and a 1-month-old infant recorded at 7 kg and 67 cm) and could not be corrected against the source documents; these records were excluded. The nutritional analyses therefore included 30 children (20 survivors and 10 non-survivors).

The median weight-for-age z-score among these 30 children was −0.91 (IQR −2.71 to 0.15). Eleven children (36.7%; 95% CI 21.5–55.0) had malnutrition (z < −2), and five (16.7%) had severe malnutrition (z < −3). We did not detect an association with mortality for malnutrition (6 of 20 survivors vs. 5 of 10 non-survivors; *p* = 0.425), severe malnutrition (2 of 20 vs. 3 of 10; *p* = 0.300), or the z-score itself (−0.84, IQR −2.35 to 0.26 vs. −2.00, IQR −3.60 to 0.01; *p* = 0.344). The weight-for-age z-score showed no detectable correlation with serum albumin (Spearman’s rho = 0.17, *p* = 0.357) or 25(OH)D (rho = 0.12, *p* = 0.512), but it correlated positively with vitamin C (rho = 0.39, *p* = 0.033; Supplementary Figure S2). In this subgroup of 30 children, the unadjusted difference in vitamin C between survivors and non-survivors was not statistically significant (73.8 vs. 51.1 µmol/L, *p* = 0.115), and the rank correlation between vitamin C and vital status weakened from −0.30 to −0.25 after partialling out the z-score.

### 3.6. Severity Scores, Organ Dysfunction, and Other Biomarkers

We did not detect differences between groups in admission severity scores (Phoenix, 3 vs. 3, *p* = 0.583; PRISM.III, 11 vs. 14, *p* = 0.242; pSOFA, 6 vs. 6, *p* = 0.661; Table 1). These null results are inconclusive and do not establish that the groups were comparable. The number of organ dysfunctions recorded on the case report form was higher in non-survivors (median 2 vs. 0, *p* = 0.021); this variable could not be reconciled with the recorded organ support data and is therefore presented descriptively, without inference (Table 1). Median procalcitonin was 20.0 ng/mL (IQR 10.4–56.7) in non-survivors and 9.2 ng/mL (IQR 3.2–23.7) in survivors (*p* = 0.107). Three values were at or above the upper limit of the assay range (two were exactly 200 ng/mL); procalcitonin was therefore treated as right-censored. In exploratory, non-prespecified analyses, non-survivors had lower platelet counts (median 85 vs. 329 × 10^3^/μL, *p* = 0.009), a higher frequency of coagulopathy (6/11 vs. 4/26, *p* = 0.038), and more frequent corticosteroid administration (7/11 vs. 6/26, *p* = 0.028; Table 1). These exploratory comparisons were not corrected for multiplicity and are presented solely to inform the design of future studies.

### 3.7. Sensitivity Analysis

Excluding the patient with the internally inconsistent Phoenix record did not alter the results: vitamin C (*p* = 0.038), 25(OH)D (*p* = 0.210), lactate (*p* = 0.918), albumin (Welch’s *t*-test, *p* = 0.945), and LAR (*p* = 0.706). Restricting the analysis to the 32 patients who met the criteria for septic shock after this exclusion likewise did not alter the results (vitamin C, *p* = 0.027; 25(OH)D, *p* = 0.238; lactate, *p* = 0.807; albumin, *p* = 0.763; LAR, *p* = 0.502).

### 3.8. Cross-Classification of Vitamin C and 25(OH)D Status (Post Hoc)

In a post hoc exploratory analysis, patients were cross-classified according to whether plasma vitamin C was below the cohort median (73.8 umol/L; all values were above the hypovitaminosis threshold) and whether 25(OH)D was <20 ng/mL. The multiplicative interaction between the two categories was not statistically significant (Firth-penalized model, Wald *p* = 0.34), and the cell sizes (6–13 children, 0–5 deaths per cell) are too small to support inference. The detailed results are reported in Supplementary Table S2 and Supplementary Figure S3 and do not establish a combined or synergistic effect of vitamins C and D.

## 4. Discussion

This single-center, exploratory, observational study examined 37 children with sepsis or septic shock admitted to the PICU. In unadjusted analysis, lower plasma vitamin C concentrations at admission were associated with mortality, whereas no association was detected for 25(OH)D, lactate, albumin, or the LAR. Three aspects of this finding require emphasis. First, all children in the cohort had vitamin C concentrations well above the accepted threshold for hypovitaminosis C (23 µmol/L), so the finding concerns relative differences within a physiologically adequate range rather than hypovitaminosis or deficiency. Second, after correction for multiple comparisons, the association was no longer statistically significant (q = 0.141), and the confidence interval for the AUC (0.55–0.90) is compatible with discrimination ranging from near chance to good. Third, vitamin C correlated with nutritional status in the children for whom weight-for-age could be validly assessed, and within that subgroup the difference between survivors and non-survivors was no longer statistically significant. Nutritional confounding therefore remains a plausible alternative explanation rather than one that has been excluded (Section 3.5 and Supplementary Figure S2). These data are hypothesis-generating and do not demonstrate prognostic utility.

In a secondary analysis of the Swiss Pediatric Sepsis Study involving 61 children, Equey et al. reported hypovitaminosis C in 36% of patients and deficiency in 10% [2]. In a study of 34 critically ill children, Choi et al. observed hypovitaminosis C (<23 µmol/L) in 47% and did not detect differences in severity scores between patients with hypovitaminosis C and those with adequate concentrations [25]. In a study of 221 children with suspected sepsis, McWhinney et al. found that concentrations below 11 µmol/L were associated with a 3.38-fold higher likelihood of two or more organ failures [27]. Fathi et al. reported a comparable prevalence of deficiency in a UK PICU [26]. The present cohort differs markedly from these series: whereas approximately one-third to one-half of the children in the European and Australian studies had hypovitaminosis C, no child in this cohort did. Several explanations merit consideration beyond sample size. First, regional dietary ascorbate intake in northwestern Mexico may exceed that of the reference populations, although intake was not measured in this study. Direct comparison between studies is further complicated by differences in analytical platforms, sample matrices, and calibration across published pediatric cohorts. The present study used heparinized plasma, which yields higher ascorbate values than the serum used in several previous series [35]. Because vitamin C was recorded to one decimal place in mg/dL, measurement resolution may also contribute.

Two internal observations bear on whether the vitamin C signal is spurious. First, the decrease in mortality across ascending vitamin C tertiles (50.0%, 18.2%, and 16.7%) is more consistent with a graded relationship than with an artifact confined to a single outlier; however, the gradient was not statistically significant. Second, the inverse correlation between vitamin C and the pSOFA score (rho = −0.39, *p* = 0.017) is directionally consistent with the relationship described by McWhinney et al. in a larger cohort [27]. Neither observation confirms the association. The absence of a detectable correlation between vitamin C and albumin argues somewhat against hemodilution as the sole explanation, but it cannot exclude it, because the pre-sampling fluid volume was not recorded. Taken together, these observations support the hypothesis that ascorbate status carries information within the adequate range, a hypothesis that adequately powered future cohorts could test.

Regarding vitamin D, the present results align with the most conservative data in the literature. In a study of 119 critically ill children, Loni et al. observed a deficiency prevalence of 47.1% and found no association with mechanical ventilation, length of hospital stay, or mortality [41]. Ebenezer et al., by contrast, reported a deficiency prevalence of 40.3% and lower concentrations in children with higher severity scores [42]. Kumar et al. found an association between vitamin D status and mortality in a much larger cohort of 522 PICU patients [10]. The present cohort had a lower prevalence of 25(OH)D < 20 ng/mL, and we did not detect a difference between survivors and non-survivors. The confidence interval for the median difference was wide enough to include clinically relevant effects in both directions (−18.8 to 2.4 ng/mL). These results indicate that this study could not detect an association, not that no association exists. Because the 2024 Endocrine Society guideline no longer endorses specific 25(OH)D thresholds [12], comparisons of “deficiency” prevalence across studies should be interpreted as comparisons of historical cut-offs rather than of clinically validated states.

The lack of an association between lactate, albumin, or the LAR and mortality diverges most clearly from the published literature. Previous studies have associated elevated admission lactate with 30-day mortality in children with suspected sepsis [21], and admission hypoalbuminemia has been linked to poor prognosis in several pediatric intensive care series [18,19,20]. Ray et al. reported that an elevated LAR was associated with mortality and multiple organ dysfunction in a large PICU cohort [23]. Moustafa et al. reported that the LAR discriminated organ dysfunction and mortality in children with severe sepsis [24], and Perez et al. linked the LAR to microcirculatory abnormalities in pediatric sepsis [22]. These data suggest a specific, testable explanation rather than a contradiction. The LAR is a ratio whose value depends on variation in both components. In this cohort, the LAR was almost perfectly collinear with lactate (rho = 0.95) and showed only a weak relationship with albumin (rho = −0.34); it therefore provided little information beyond lactate itself. Admission lactate was normal in most patients (median 1.30 mmol/L; 11 of 37 had values above 2.5 mmol/L), and albumin concentrations were virtually identical across groups. Notably, the published cohorts in which the LAR performed well were characterized by more pronounced hyperlactatemia and higher mortality. In the prospective Vietnamese cohort reported by Le et al., 28-day mortality was 63.4%, and the optimal LAR threshold was 1.84, more than three times the 0.5 threshold cited in the pediatric literature [28]; in the Egyptian cohort, the optimal admission cut-off was 1.17 [24]. These observations suggest that the prognostic value of the LAR and the applicability of the 0.5 threshold may depend on hyperlactatemia, and that neither can be assumed to apply to children presenting with normolactatemia at admission. This hypothesis is generated by the present data, not established by them.

A lower *p*-value for one marker than for another does not demonstrate superiority. Such a claim requires a formal comparison of discriminatory power and an assessment of incremental value relative to the Phoenix or PRISM III scores, together with calibration and validation, none of which is feasible with 11 events. The exploratory observation that non-survivors had markedly lower platelet counts is nonetheless consistent with a large body of evidence linking sepsis-associated thrombocytopenia and coagulopathy to mortality in children. Because these comparisons were exploratory and not corrected for multiplicity, we present them only as a direction for future research.

Three features of this study merit comment, each with its own limitations. To our knowledge, this is the first Latin American pediatric sepsis cohort in which vitamin C, 25(OH)D, lactate, albumin, and the LAR were measured together in prospectively enrolled children classified according to the 2024 Phoenix criteria. Measuring the five markers within the same cohort reduces between-study heterogeneity in population, timing, and assay methods and allows the markers to be examined under comparable conditions; it does not, however, eliminate confounding, and residual confounding by nutritional status, disease severity, and pre-admission fluid exposure remains possible (Section 4.1). It is also, to our knowledge, the first reported pediatric sepsis cohort in which no child had hypovitaminosis C, which makes it possible to ask whether ascorbate status carries information within the adequate range, although the cohort is too small to answer that question. Finally, the study provides a prospective evaluation of the 0.5 LAR threshold in children classified by the Phoenix criteria; in this small cohort with predominantly normal admission lactate, the threshold did not demonstrate discriminatory ability. Given the width of the confidence intervals and the small number of events, this null result does not establish that the threshold lacks discriminatory value; it identifies normolactatemia as a boundary condition that the existing literature, derived from hyperlactatemic populations with high mortality, does not address.

### 4.1. Limitations

Several limitations require caution in interpreting these results. (i) The cohort was small (37 patients, 11 deaths) and the study was single-center; with approximately one event per candidate predictor, multivariable adjustment was not possible, and only very large effects could be detected (minimum detectable Cohen’s d = 1.04). (ii) The design was observational, so residual confounding related to disease severity, nutrition, duration of sepsis before sampling, and pre-admission fluid status cannot be excluded. (iii) The outcome was PICU mortality; 28-day mortality and time-to-event analyses were not available, and biomarkers were measured only once, at admission, so trajectories could not be assessed. (iv) Ascorbate was analyzed within 48 h in refrigerated, light-protected, heparinized plasma rather than in acid-stabilized frozen aliquots, so absolute values may not be transferable between laboratories; concentrations were reported to a resolution of 0.1 mg/dL (5.7 umol/L), which produced numerous ties, and we did not have access to the assay performance parameters, so an analytical explanation for the uniformly high values cannot be excluded. (v) 25(OH)D was measured using a point-of-care immunoassay rather than liquid chromatography–tandem mass spectrometry, which could introduce method-dependent bias; moreover, the 25(OH)D categories used are historical cut-offs that are no longer endorsed by the Endocrine Society [12]. (vi) Comorbidities, the source of infection, culture results, fluid volume before sampling, exogenous albumin, and nutritional support were not recorded, so the case mix, the causative pathogens, and the appropriateness of antimicrobial treatment cannot be described. (vii) Neither age-adjusted mean arterial pressure nor the individual Phoenix subscores were recorded; the cardiovascular subscore was therefore based solely on vasoactive support and lactate, which can underestimate shock severity. (viii) The recorded count of organ dysfunctions could not be reconciled with the organ support data, and procalcitonin was right-censored at the upper limit of the assay range; neither variable supports inference. (ix) No thresholds were derived in this study, and exploratory analyses were not corrected for multiplicity. (x) No statistical analysis plan was registered before data analysis; the family of five principal comparisons and the sensitivity analyses were defined during analysis and peer review and should not be regarded as prespecified.

The most important limitation concerns anthropometry, because nutrition is the most plausible confounder of the relationship between micronutrients and mortality. The measurements were derived from routine records: one weight was corrected against the source document, two biologically implausible records were excluded, five children exceeded the 60-month limit of the WHO 2006 standards (four of whom also exceeded the 120-month limit of the WHO 2007 weight-for-age reference), and the z-score variable held in the database was not reproducible and was replaced by recalculated values. Only weight-for-age could be calculated, which does not distinguish wasting from stunting. Among the 30 evaluable children, we detected no difference in this indicator by outcome and no correlation with albumin or 25(OH)D, but a correlation with vitamin C (Supplementary Figure S2). In this subgroup, the vitamin C difference was no longer statistically significant. Nutritional confounding of the ascorbate finding therefore cannot be excluded. With only ten events, this question could not be resolved in the present study, and it remains a priority for future confirmatory research.

### 4.2. Generalizability

These findings should be extrapolated with caution. The cohort was recruited at a single tertiary pediatric referral center in northwestern Mexico and consisted predominantly of infants, with a median age of 10 months. It had a high proportion of septic shock (89.2%) and high PICU mortality (29.7%), which exceeds the mortality reported in most pediatric sepsis cohorts from high-income countries, but is consistent with the 28.5% in-hospital mortality reported for children with Phoenix-defined sepsis in resource-limited settings [29]. In contrast to the European and Australian series, vitamin C status was adequate in this population, so the vitamin C results cannot be extrapolated to populations with a substantial prevalence of hypovitaminosis C.

Conversely, the null findings for lactate, albumin, and the LAR were obtained in a cohort with low admission lactate and near-normal albumin concentrations, and they may therefore not apply to populations with more severe hyperlactatemia or hypoalbuminemia.

## 5. Conclusions

This exploratory, single-center prospective cohort study evaluated five admission biomarkers in children with sepsis and septic shock defined by the Phoenix criteria. In unadjusted analysis, non-survivors had lower plasma vitamin C concentrations at PICU admission than survivors; all values lay above the accepted threshold for hypovitaminosis C, the difference did not persist after correction for multiple comparisons, and vitamin C correlated with the weight-for-age z-score; therefore, the possibility of nutritional confounding factors cannot be ruled out. Nutritional confounding cannot be excluded. No association with mortality was detected for 25(OH)D, arterial lactate, albumin, or the lactate-to-albumin ratio, and no discrimination was detected for the proposed LAR threshold of 0.5 in this small cohort with predominantly normal admission lactate. These data establish neither independent prognostic value nor superiority of any marker, support no causal inference, and do not justify any change in clinical practice. Adequately powered multicenter cohorts are required, with a fully documented and validated preanalytical and analytical protocol for ascorbate, prospective measurement of anthropometry and dietary intake, serial sampling, a registered statistical analysis plan, and prespecified, time-defined mortality endpoints, to determine whether micronutrient status carries prognostic information in pediatric sepsis and whether the prognostic value of the lactate-to-albumin ratio depends on hyperlactatemia.

## Figures and Tables

**Table 1:** Clinical, demographic, and biochemical characteristics of the 37 children studied, by vital status at PICU discharge.

Variable	Total (*n* = 37)	Survivors (*n* = 26)	Non-Survivors (*n* = 11)	*p*
Age, months, median (IQR)	10 (3–24)	12.0 (3.2–24.0)	9.0 (2.5–11.0)	0.270
Male sex, *n* (%)	23 (62.2)	16 (61.5)	7 (63.6)	1.000
Weight-for-age z-score, median (IQR) ^a^	−0.91 (−2.71 to 0.15)	−0.84 (−2.35 to 0.26)	−2.00 (−3.60 to 0.01)	0.344
Malnutrition (z < −2), *n* (%) ^a^	11/30 (36.7)	6/20 (30.0)	5/10 (50.0)	0.425
Severe malnutrition (z < −3), *n* (%) ^a^	5/30 (16.7)	2/20 (10.0)	3/10 (30.0)	0.300
Septic shock (Phoenix ≥ 2 + ≥1 CV point), *n* (%)	33 (89.2)	23 (88.5)	10 (90.9)	1.000
Plasma vitamin C, mg/dL, median (IQR)	1.30 (0.90–1.50)	1.30 (1.12–1.65)	0.90 (0.90–1.20)	0.028
Plasma vitamin C, µmol/L, median (IQR)	73.8 (51.1–85.2)	73.8 (63.9–93.7)	51.1 (51.1–68.1)	0.028
Vitamin C < 23 µmol/L, n (%)	0 (0)	0 (0)	0 (0)	–
25(OH)D, ng/mL, median (IQR)	27.7 (17.7–39.6)	28.8 (19.3–46.6)	20.0 (15.9–28.8)	0.184
25(OH)D > 30 ng/mL, *n* (%) ^e^	14 (37.8)	12 (46.2)	2 (18.2)	0.256
25(OH)D 20–30 ng/mL, *n* (%) ^e^	11 (29.7)	6 (23.1)	5 (45.5)	
25(OH)D < 20 ng/mL, *n* (%) ^e^	12 (32.4)	8 (30.8)	4 (36.4)	
Procalcitonin, ng/mL, median (IQR) ^b^	12.1 (4.4–39.0)	9.2 (3.2–23.7)	20.0 (10.4–56.7)	0.107
Arterial lactate, mmol/L, median (IQR)	1.30 (0.90–3.00)	1.30 (1.00–2.98)	1.50 (0.80–2.85)	0.894
Serum albumin, g/dL, mean (SD)	3.34 (0.73)	3.34 (0.77)	3.36 (0.69)	0.916
Lactate-to-albumin ratio, median (IQR)	0.37 (0.29–0.74)	0.35 (0.29–0.73)	0.48 (0.29–0.80)	0.752
LAR > 0.5, *n* (%)	15 (40.5)	10 (38.5)	5 (45.5)	0.728
Phoenix Sepsis Score, median (IQR)	3 (2–4)	3 (2–4)	3 (3–4)	0.583
Phoenix cardiovascular subscore, median (IQR)	2 (1–2)	2 (1–2)	2 (1–2)	0.554
PRISM III, median (IQR)	11 (9–14)	11.0 (8.5–12.0)	14.0 (9.5–15.5)	0.242
pSOFA, median (IQR)	6 (5–7)	6 (5–7)	6.0 (3.0–8.5)	0.661
Recorded organ dysfunctions, *n*, median (IQR) ^c^	0 (0–2)	0 (0–2)	2 (1–3)	0.021
Vasoactive agents—none, *n* (%)	4 (10.8)	3 (11.5)	1 (9.1)	0.341
Vasoactive agents—one, *n* (%)	12 (32.4)	9 (34.6)	3 (27.3)	
Vasoactive agents—two or more, *n* (%)	21 (56.8)	14 (53.8)	7 (63.6)	
Vasoactive–inotropic score, median (IQR)	20 (8–40)	10.5 (8.2–25.0)	40.0 (12.5–81.0)	0.106
Invasive mechanical ventilation, *n* (%)	35 (94.6)	25 (96.2)	10 (90.9)	0.512
Duration of ventilation among ventilated children, days, median (IQR) ^d^	8 (4–12); *n* = 35	7.5 (4.0–11.5); *n* = 25	9.0 (4.5–24.0); *n* = 10	0.752
Renal replacement therapy, *n* (%)	4 (10.8)	3 (11.5)	1 (9.1)	1.000
Coagulopathy, *n* (%)	10 (27.0)	4 (15.4)	6 (54.5)	0.038
Platelets, ×10^3^/µL, median (IQR)	183 (71–372)	329 (133–425)	85 (46–107)	0.009
Hypoglycaemia, *n* (%)	4 (10.8)	1 (3.8)	3 (27.3)	0.070
Corticosteroid administration, *n* (%)	13 (35.1)	6 (23.1)	7 (63.6)	0.028
PICU length of stay, days, median (IQR)	22 (11–52)	23 (16–50)	11 (9–46)	0.134

IQR: interquartile range. SD: standard deviation. CV: cardiovascular. PRISM: Pediatric Risk of Mortality. pSOFA: Pediatric Sequential Organ Failure Assessment. LAR: lactate-to-albumin ratio. Continuous variables were compared using the Mann–Whitney U test, except for albumin, for which Welch’s *t*-test was used (Shapiro–Wilk *p* = 0.37 in both groups). Categorical variables were compared using Fisher’s exact test, except for the three-level 25(OH)D category, which was compared using a Monte Carlo exact test with 100,000 replicates. The *p*-value shown for vasoactive agents corresponds to the Mann–Whitney U test applied to the number of vasoactive agents (range 0–5). All percentages use the denominator indicated in the corresponding column header, unless a different denominator is stated explicitly in the row. One patient (2.7%) was excluded from the analyses of serum calcium and phosphorus because these values were missing. Preexisting comorbidities, anatomical sources of infection, and microbiological culture results were not recorded (Section 2.4). ^a^ Weight-for-age z-scores were recalculated from age, sex, and weight using the WHO Child Growth Standards (2006) [33]; denominators are total *n* = 30, survivors *n* = 20, non-survivors *n* = 10, because five children were older than 60 months (the upper age limit of the WHO 2006 standards; four of these children were also older than 120 months) and two records contained biologically implausible anthropometric values (Section 3.5). ^b^ Procalcitonin values show a pattern consistent with reporting at the upper limit of the assay range; the reporting range could not be documented, and no inference is drawn. ^c^ The case report form presents the count of organ dysfunctions descriptively only, and no inference is drawn from it (Section 3.6). ^d^ Limited to the 35 children who received invasive mechanical ventilation. ^e^ Historical cut-offs commonly used in previous pediatric studies (2011 Endocrine Society guideline); they are not endorsed by the 2024 Endocrine Society guideline and are used for descriptive comparison only.

**Table 2:** Effect estimates with 95% confidence intervals and false-discovery-rate correction for the five principal biomarker comparisons.

Biomarker	Effect Estimate (95% CI) ^a^	AUC (95% CI)	Firth OR (95% CI) ^b^	*p*	q ^c^
Plasma vitamin C, µmol/L	−17.0 (−28.4 to −5.7)	0.73 (0.55–0.90)	0.63 (0.37–0.94) per 10 µmol/L	0.028	0.141
25(OH)D, ng/mL	−6.9 (−18.8 to 2.4)	0.64 (0.45–0.82)	0.76 (0.43–1.09) per 10 ng/mL	0.184	0.459
Arterial lactate, mmol/L	+0.10 (−0.60 to 1.10)	0.52 (0.30–0.73)	1.01 (0.77–1.29) per 1 mmol/L	0.894	0.916
Serum albumin, g/dL	+0.03 (−0.51 to 0.56)	0.50 (0.31–0.69)	1.05 (0.41–2.71) per 1 g/dL	0.916	0.916
Lactate-to-albumin ratio	+0.03 (−0.19 to 0.31)	0.53 (0.32–0.74)	1.00 (0.93–1.06) per 0.1 unit	0.752	0.916

AUC: area under the receiver operating characteristic curve. CI: confidence interval. OR: odds ratio. ^a^. Hodges–Lehmann median difference (non-survivors minus survivors) with distribution-free exact limits, except for albumin, for which the Welch mean difference is reported. ^b^. Unadjusted Firth-penalized odds ratio for PICU mortality with profile penalized-likelihood confidence limits, calculated for all five principal biomarkers irrespective of the result of the unadjusted comparison. ^c^. Benjamini–Hochberg false discovery rate across the five comparisons. All estimates are exploratory and unadjusted.

## Data Availability

The de-identified dataset analyzed during the current study is available from the corresponding author upon reasonable request, subject to approval by the research ethics committee of the Pediatric Hospital of Sinaloa.

## Supplementary Materials

The following supporting information can be downloaded at: https://www.mdpi.com/article/10.3390/pediatric18050132/s1, Figure S1: Participant flow diagram. Figure S2: Relationship between the weight-for-age z-score and plasma vitamin C concentration at PICU admission in the 30 children for whom a valid weight-for-age score could be assigned. Figure S3: PICU mortality by post hoc cross-classification of plasma vitamin C (below vs. at or above the cohort median of 73.8 umol/L) and 25(OH)D (<20 vs. ≥20 ng/mL). Table S1: STROBE Statement-Checklist of items for cohort studies. Table S2: PICU mortality by post hoc cross-classification of plasma vitamin C and 25(OH)D status.
