# Supplementary material for: Plasma Vitamin C, 25-Hydroxyvitamin D, and the Lactate-to-Albumin Ratio at PICU Admission in Survivors and Non-Survivors of Phoenix-Defined Pediatric Sepsis and Septic Shock: An Exploratory Prospective Cohort Study
Source: Pediatr Rep. 2026 Oct 2;18(5):132. doi: 10.3390/pediatric18050132 (PMC13708177; doi:10.3390/pediatric18050132)
Supplement: Supplementary file 1 [file pediatrrep-18-00132-s001.zip › pediatrrep-4554840-supplementary.pdf]

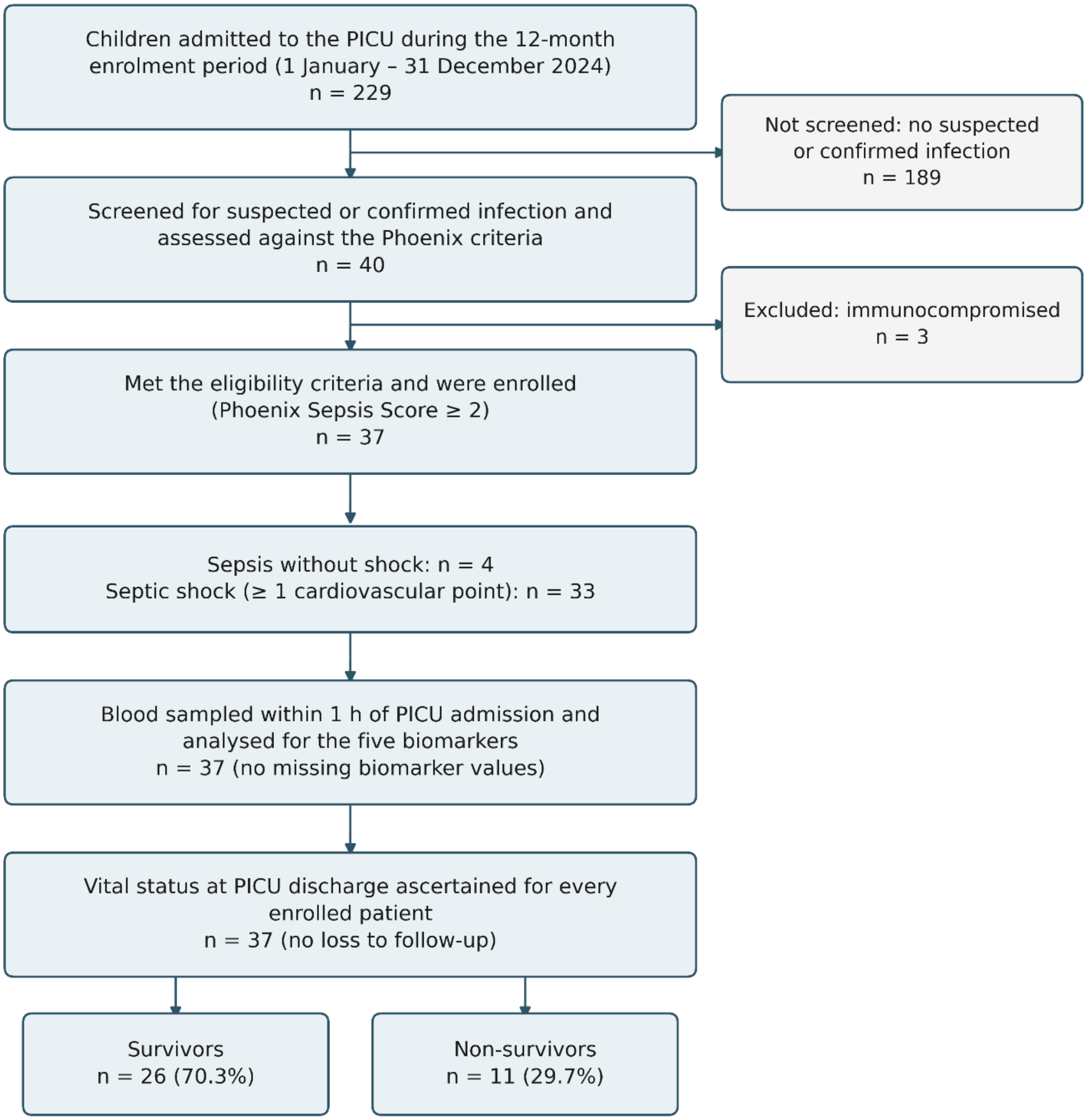

Supplementary Figure S1. Participant flow diagram.

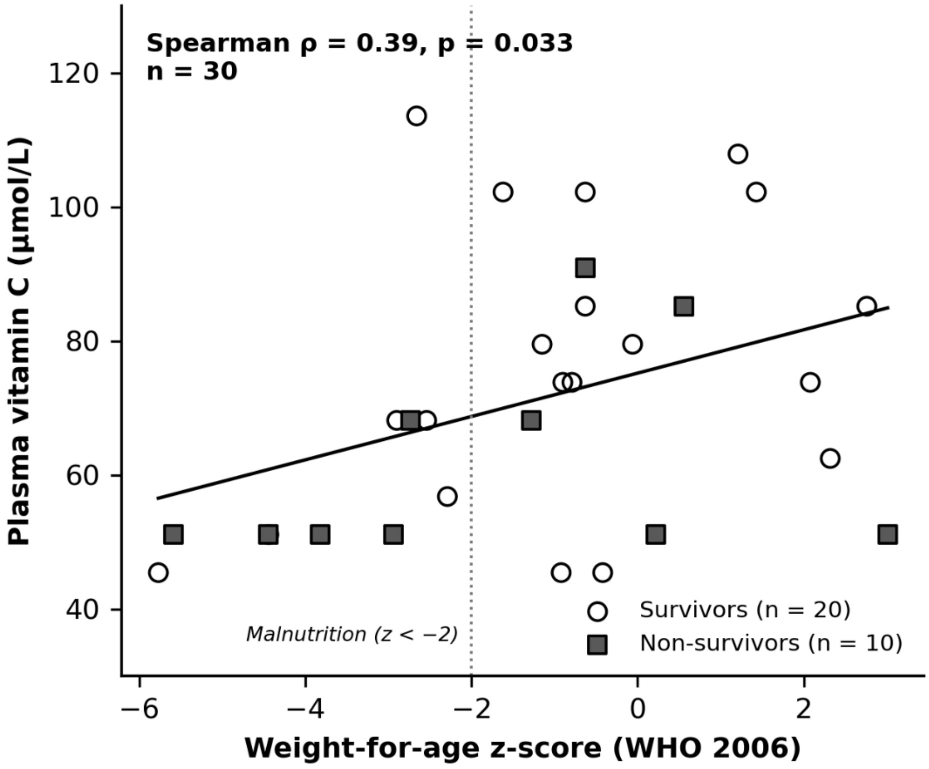

Supplementary Figure S2. Relationship between the weight-for-age z-score and plasma vitamin C concentration at PICU admission in the 30 children for whom a valid weight-for-age score could be assigned. Hollow circles represent survivors (n = 20) and filled squares represent non-survivors (n = 10). The solid line is the least-squares fit, and the vertical dashed line marks the malnutrition threshold (z < -2). Weight-for-age z-scores were calculated from age, sex, and weight using the WHO Child Growth Standards (2006). Spearman's rho = 0.39; p = 0.033. The horizontal distribution of the data points reflects the granularity of the vitamin C measurement (0.1 mg/dL = 5.7 umol/L).

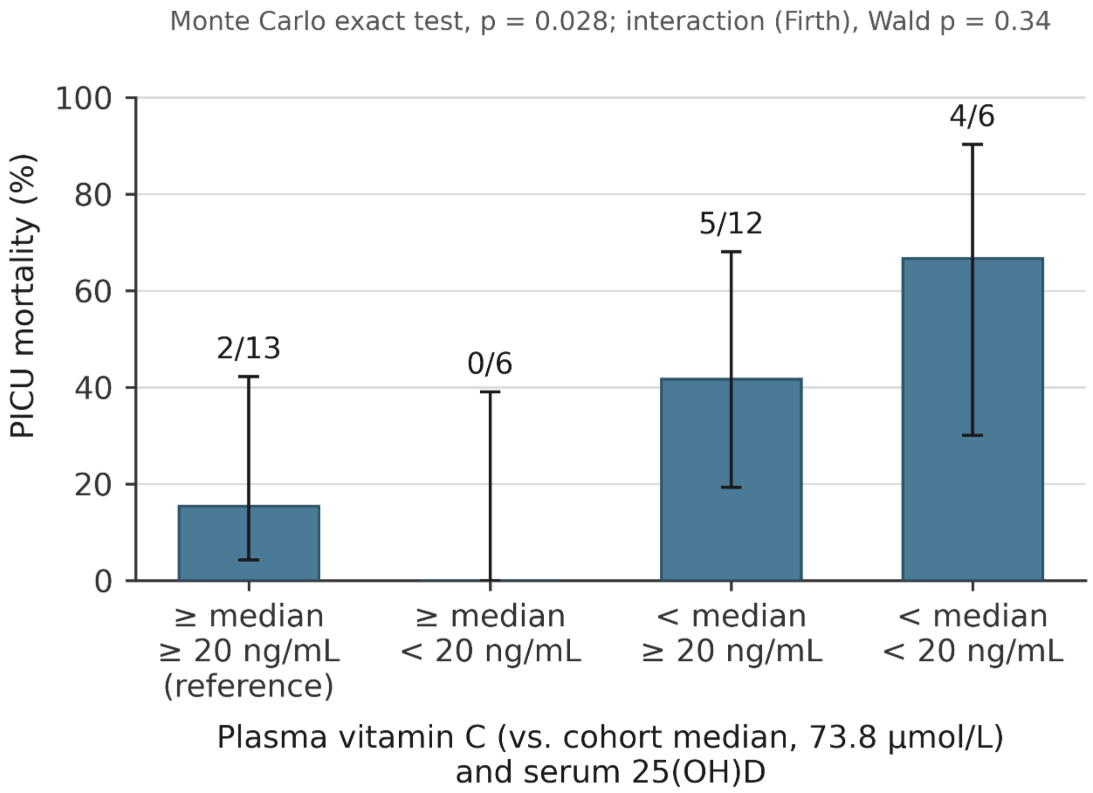

Supplementary Figure S3. PICU mortality by post hoc cross-classification of plasma vitamin C (below vs. at or above the cohort median of 73.8 umol/L) and 25(OH)D (< 20 vs. ≥ 20 ng/mL). All vitamin C values were above the hypovitaminosis threshold; "low vitamin C" therefore denotes a concentration below the cohort median, not hypovitaminosis or deficiency. The multiplicative interaction was not statistically significant (Firth-penalized model, Wald p = 0.34), and the cell sizes are too small to support inference. Detailed counts are given in Supplementary Table S1.

Supplementary Table S1. STROBE Statement-Checklist of items for cohort studies

Manuscript: *Plasma Vitamin C, 25-Hydroxyvitamin D, and the Lactate-to-Albumin Ratio at PICU Admission in Survivors and Non-Survivors of Phoenix-Defined Pediatric Sepsis and Septic Shock: An Exploratory Prospective Cohort Study*

Section/Topic	Item No	Recommendation	Reported in (section / table / figure)
**Title and abstract**	1	(a) Indicate the study's design with a commonly used term in the title or the abstract	Title; Abstract (Methods)
		(b) Provide in the abstract an informative and balanced summary of what was done and what was found	Abstract
**Introduction**			
**Background/rationale**	2	Explain the scientific background and rationale for the investigation being reported	Introduction, paragraphs 1–4
**Objectives**	3	State specific objectives, including any prespecified hypotheses	Introduction, final paragraph; Section 2.6 (no prespecified primary hypothesis)
**Methods**			
**Study design**	4	Present key elements of study design early in the paper	Section 2.1
**Setting**	5	Describe the setting, locations, and relevant dates, including periods of recruitment, exposure, follow-up, and data collection	Section 2.1 (1 January–31 December 2024); Section 2.3 (follow-up to PICU discharge); Section 2.5 (sampling within 1 h of admission)
**Participants**	6	(a) Give the eligibility criteria, and the sources and methods of selection of participants. Describe methods of follow-up	Section 2.2; Section 2.3
		(b) For matched studies, give matching criteria and number of exposed and unexposed	Not applicable (unmatched cohort)
**Variables**	7	Clearly define all outcomes, exposures, predictors, potential confounders, and effect modifiers. Give diagnostic criteria, if applicable	Sections 2.2 (Phoenix criteria, cardiovascular component), 2.3 (outcome), 2.4 (clinical variables, unavailable variables), 2.5 (biomarkers, thresholds)
**Data sources/measurement**	8*	For each variable of interest, give sources of data and details of methods of assessment (measurement). Describe comparability of assessment methods if there is more than one group	Sections 2.4 and 2.5 (matrices, preanalytical handling, analytical methods, unit conversion); identical handling for both groups (Section 2.5)
**Bias**	9	Describe any efforts to address potential sources of bias	Section 2.2 (consecutive enrollment); Section 2.4 (timing of sampling); Section 2.5 (laboratory staff blinded to outcome, uniform handling); Section 4.1
**Study size**	10	Explain how the study size was arrived at	Section 2.6 (all eligible patients over a fixed 12-

			month period; post hoc minimum detectable effect)
**Quantitative variables**	11	Explain how quantitative variables were handled in the analyses. If applicable, describe which groupings were chosen and why	Section 2.5 (reference thresholds, unit conversion); Section 2.6; Section 3.2 (tertiles given as observed ranges)
**Statistical methods**	12	(a) Describe all statistical methods, including those used to control for confounding	Section 2.6 (no multivariable model; reasons stated)
		(b) Describe any methods used to examine subgroups and interactions	Section 2.6; Sections 3.4, 3.5, and 3.8
		(c) Explain how missing data were addressed	Section 2.6 (complete-case analysis)
		(d) If applicable, explain how loss to follow-up was addressed	Sections 2.3 and 3.1 (vital status ascertained for all patients; no loss to follow-up)
		(e) Describe any sensitivity analyses	Section 2.6; Section 3.7
**Results**			
**Participants**	13*	(a) Report numbers of individuals at each stage of study—eg numbers potentially eligible, examined for eligibility, confirmed eligible, included in the study, completing follow-up, and analysed	Section 3.1; Supplementary Figure S1
		(b) Give reasons for non-participation at each stage	Section 3.1; Supplementary Figure S1
		(c) Consider use of a flow diagram	Supplementary Figure S1
**Descriptive data**	14*	(a) Give characteristics of study participants (eg demographic, clinical, social) and information on exposures and potential confounders	Section 3.1; Table 1
		(b) Indicate number of participants with missing data for each variable of interest	Section 2.6; Section 3.5; Table 1 (footnotes)
		(c) Summarise follow-up time (eg, average and total amount)	Section 3.1 (median 22 days, IQR 11–52)
**Outcome data**	15*	Report numbers of outcome events or summary measures over time	Section 3.1 (11 deaths; 29.7%); Table 1
**Main results**	16	(a) Give unadjusted estimates and, if applicable, confounder-adjusted estimates and their precision (eg, 95% confidence interval). Make clear which confounders were adjusted for and why they were included	Sections 3.2–3.4; Table 2; Figures 1–5 (unadjusted estimates only; adjustment not feasible, Section 2.6)
		(b) Report category boundaries when continuous variables were categorized	Sections 2.5 and 3.2; Figure 2
		(c) If relevant, consider translating estimates of relative risk into absolute risk for a meaningful time period	Section 3.2 and Figure 2 (mortality by tertile); Section 3.4 (mortality by LAR threshold)
**Other analyses**	17	Report other analyses done—eg analyses of subgroups and interactions, and sensitivity analyses	Sections 3.4–3.8; Supplementary Figures S2–S3; Supplementary Table S1
**Discussion**			
**Key results**	18	Summarise key results with reference to study objectives	Discussion, first paragraph

**Limitations**	19	Discuss limitations of the study, taking into account sources of potential bias or imprecision. Discuss both direction and magnitude of any potential bias	Section 4.1
**Interpretation**	20	Give a cautious overall interpretation of results considering objectives, limitations, multiplicity of analyses, results from similar studies, and other relevant evidence	Discussion; Conclusions
**Generalisability**	21	Discuss the generalisability (external validity) of the study results	Section 4.2
**Other information**			
**Funding**	22	Give the source of funding and the role of the funders for the present study and, if applicable, for the original study on which the present article is based	Funding statement (no funding)

*Give information separately for exposed and unexposed groups (here: survivors and non-survivors) where applicable.

Checklist items are from the STROBE Statement for cohort studies (von Elm et al., Lancet 2007;370:1453–1457; Vandenbroucke et al., PLoS Med 2007;4:e297). Locations refer to the revised manuscript.

Supplementary Table S2. PICU mortality by post hoc cross-classification of plasma vitamin C and 25(OH)D status.

Group	n	Deaths	Mortality, % (95% CI) [a]
Vitamin C ≥ cohort median and 25(OH)D ≥ 20 ng/mL (reference)	13	2	15.4 (4.3–42.2)
Vitamin C ≥ cohort median and 25(OH)D < 20 ng/mL	6	0	0 (0–39.0)
Vitamin C < cohort median and 25(OH)D ≥ 20 ng/mL	12	5	41.7 (19.3–68.0)
Vitamin C < cohort median and 25(OH)D < 20 ng/mL	6	4	66.7 (30.0–90.3)

Post hoc exploratory analysis. Cohort median of plasma vitamin C = 1.3 mg/dL (73.8 µmol/L); all values were above the hypovitaminosis threshold (<23 µmol/L), so "vitamin C < cohort median" does not denote hypovitaminosis or deficiency. The 25(OH)D cut-off of 20 ng/mL is a historical cut-off (see Section 2.5). Overall comparison, Monte Carlo exact test (100,000 replicates), $p = 0.028$; multiplicative interaction in a Firth-penalized logistic model, Wald $p = 0.34$. Cell sizes are too small to support inference, and the data do not establish a combined or synergistic effect. [a] Wilson 95% confidence interval.
